# Why the Medicare physician fee schedule misvalues fee levels and how to fix it

**DOI:** 10.1093/haschl/qxaf189

**Published:** 2025-10-01

**Authors:** Laura Skopec, Robert A Berenson

**Affiliations:** Health Policy Division, Urban Institute, 500 L’Enfant Plaza SW, Washington DC 20024, United States; Health Policy Division, Urban Institute, 500 L’Enfant Plaza SW, Washington DC 20024, United States

**Keywords:** Medicare, physician payment, Medicare Physician Fee Schedule, relative values

## Abstract

The Centers for Medicare and Medicaid Services (CMS) relies on the American Medical Association's Relative Value Scale Update Committee (RUC) to estimate the physician work and direct practice expense associated with the Medicare Physician Fee Schedule (MPFS). However, as CMS notes in the 2026 MPFS proposed rule, the RUC's processes, which rely heavily on surveys and expert panels of physicians who are members of specialty societies, create conflicts of interest and overvalue specialty services. Although CMS and the RUC regularly assess MPFS codes for misvaluation, significant distortions remain, in part because the RUC develops new values by simply repeating the survey and expert panel processes that created the misvaluation in the first place. To correct this longstanding program, CMS should implement a technical expert panel to provide unbiased recommendations on the fee schedule, and Congress should require CMS to validate work and direct practice expense values using alternative, empirical data sources.

## Introduction

The Centers for Medicare and Medicaid Services (CMS) is responsible for maintaining the Medicare Physician Fee Schedule (MPFS), which sets prices for thousands of Healthcare Common Procedure Coding System codes billed by physicians and other clinicians (HCPCS codes include the Current Procedural Terminology (CPT) codes trademarked by the American Medical Association). Medicare currently approves about $90 billion in MPFS allowed charges annually.^1^ The MPFS is also widely used by Medicare Advantage insurers, state Medicaid programs, and commercial insurers to set physician fees in their own fee schedules, such that the MPFS influences over $1 trillion in US healthcare spending for clinician services.^2^

MPFS fees are based on the Resource-Based Relative Value System (RBRVS), which assigns work, practice expense (PE), and professional liability insurance (PLI) relative value units (RVUs) to each of the physician-billed codes Medicare covers (see Supplementary Appendix). CMS relies on the American Medical Association's (AMA's) Relative Value Scale Update Committee (RUC) to help determine appropriate values for work RVUs and direct PE (eg, clinical labor, medical equipment, and disposable supplies) for each code. The RUC consists of 32 members, 22 of whom are appointed by medical specialty societies.^3^ The RUC develops recommendations for work RVUs by surveying the physicians who bill the service through their specialty societies. The RUC develops direct PE RVU recommendations primarily through expert panels.

Because the relative resources needed to provide a service can change over time due to changes in practice patterns or technology, ensuring the accuracy of relative values requires frequent review.^4^ Accurate relative values are the building blocks for credible fees in the MPFS. If payment deviates substantially from the resources needed to provide the service, it can lead to excessive provision of overvalued services that may be clinically inappropriate while undervalued services may be short-changed. Distorted fees also send signals to younger physicians about which specialties to pursue, with one association predicting a shortage of up to 40 000 primary care physicians by 2036.^5^

## How the RUC and CMS develop RVUs

CMS is statutorily responsible for establishing and revising RVUs, but it coordinates closely with the RUC, especially for work and direct PE. In its 2015 report on Medicare physician payment, the Government Accountability Office (GAO) provided a detailed description of the process for establishing RVUs that we believe is still in effect, and we summarize it in the remainder of this section.^4^

There are three main steps in the process to develop and establish RVUs: (1) CMS, the RUC, and the AMA's CPT editorial panel identify services for RUC review; (2) the RUC works with specialty societies to field surveys and develop and revise work relative values and direct PE recommendations for identified services; and (3) CMS reviews each RUC recommendation and engages in notice and comment rulemaking to establish RVUs.^4^

When new codes are added or existing codes are identified for review, the RUC works with relevant specialty societies to see which services they want to develop recommendations for, and to determine whether there are related services in the same family that should also be considered for review.^4^ Societies may also assert that the current values are accurate.

Specialty societies then develop work RVU recommendations through a member survey process. The specialty societies use the RUC survey instrument to ask a sample of their members about the time needed to perform the service, the complexity and intensity of performing that service relative to a reference service, and the total estimated work value of the service. The respondents select a reference service from a list of 10-15 comparable services,^4^ which may include services outside of the members' specialty that they do not typically perform (per authors' conversation with a former RUC member). Specialty societies also provide detailed descriptions of the work encompassed by each code to help orient RUC members to the likely intensity of each code. These work descriptions provide an opportunity for specialty societies to exaggerate the work entailed in the codes they review; some do.^6^

Specialty societies then evaluate the survey results to determine whether they are consistent with the relative values of other services with well-established RVUs, a process called “magnitude estimation.” Specialty societies can make recommendations that differ from their survey results when those results are inconsistent with other services in the MPFS.

For direct PE, specialty societies rely primarily on expert panels to estimate expenses like clinical labor, medical equipment, and disposable supplies. Because of inconsistencies across services and specialty societies, some preservice and postservice components of direct PE have been standardized for major procedures, such as dress, scrub, and wait time (per authors' conversation with a former RUC member). The RUC and specialty societies may also provide equipment and supply invoices to CMS for review and potential inclusion in direct PE calculations.

Recommendations for both work and direct PE, if applicable, are then submitted to the RUC for consideration, along with documentation. RUC members vote on each work recommendation, and those receiving a two-thirds majority are forwarded to CMS for consideration.^4^ Any direct PE recommendations are considered for each work recommendation that passes, and the RUC generally approves direct PE recommendations without modification (per authors' conversation with a former RUC member).

CMS reviews the recommendations it receives from the RUC and publishes both the RUC recommendations and any CMS-proposed alternatives in a notice of proposed rulemaking in the Federal Register. CMS may use other data sources, such as claims data, invoices, or data from the Veterans Health Administration, to review RUC work and direct PE recommendations and propose alternatives.^7^ Since 2010, CMS has accepted 85% of RUC work RVU recommendations,^8^ but no data is available on CMS acceptance of direct PE recommendations.

## Challenges in estimating work RVUs

Not all time spent on patient care is equivalent—eg, a minute of brain surgery is not equivalent to a minute interpreting a sonogram. This creates the major challenges for estimating work.^9^ Deciding how much intensity varies for 9000+ codes has long been a major challenge for achieving accurate work RVUs.

A longstanding criticism of the MPFS is that it rewards procedures and technology-heavy services more generously than office visits and other evaluation and management (E/M) services.^10^ The higher work values set for treatment, procedures, and technology-heavy services produce large payment disparities across specialties.^11^ Because primary care physicians and certain other specialties bill mostly E/M services, the MFPS results in inappropriately lower pay for these so-called “cognitive” specialties and higher pay for “procedural” specialties.^12^

## How the RUC specialty survey and magnitude estimation processes create misvalued codes

Although the specialty society surveys gather estimates of time to perform services and the complexity and intensity of the service, the RUC's recommendations focus on magnitude estimation rather than separately considering time and intensity as distinct components that comprise work. This approach was used in the Harvard study on which the RBRVS system is based because the researchers could not gather empirical time and intensity data for thousands of codes.^13^ However, in contrast to physicians' voluntary participation in the Harvard research study, the RUC's use of magnitude estimation relies on conflicted participants who know that their estimates will affect their own and their colleagues' incomes. William Hsiao, the lead investigator of the original Harvard study, said of the RUC's process, “The AMA fought very hard to take over this updating process. I said this had to be done by an impartial group of people. This is highly political.”^14^

The GAO has found weaknesses with RUC survey data, including low response rates, low total number of responses, and large ranges in responses, all of which undermine the accuracy of the RUC recommendations.^4^ They concluded, “the RUC's process for developing relative value recommendations relies on the input of physicians who may have potential conflicts of interest with respect to the outcomes of CMS's process.”^4^ Recently, CMS agreed in its 2026 MPFS proposed rule, citing “inherent conflicts of interest” and “overinflated” valuations as justification for implementing a blanket efficiency adjustment to non-time-based codes.^7^

The AMA maintains that the RUC members function as a panel of experts, rather than as advocates for their own specialty society's codes. That is, they are endorsed by the AMA as impartial judges of physician work and direct PE. Yet, a thorough review detailing problems with the RUC's processes, supported by current and former RUC members we talked to on background, illustrates that the RUC functions more as a political body, not as neutral judges.^15^ RUC members typically participate in their specialty society's RVU committees in addition to the RUC, although they are prohibited from participating in the RUC discussion on their society's recommendations.^15^ An anonymous RUC member noted that RUC members commonly fail to comment on overvalued codes performed by other societies, “because to do so would invite retaliation and scrutiny of the codes their members use.”^15^ In short, RUC members engage in strategic behavior related to the services their society's recommendations forwards to the RUC.

## History of the potentially misvalued code initiative

By law, CMS must review and, if needed, update the MPFS not less than every 5 years to ensure the relative values stay up-to-date.^16^ CMS began its first 5-year review in 1994, with assistance from the RUC.^17^ In most sectors of the economy, increased efficiency and new technology allow workers to complete tasks more quickly. Productivity gains should also occur for many medical services covered by the MPFS. However, during CMS's first 5-year review of the MPFS, the RUC recommended decreases for just 10.6% of codes reviewed; nearly 90% of codes were increased or remained the same.^18^ In the second 5-year review, the RUC recommended decreases for just 3.6% of codes it reviewed. As one paper put it, RVUs “defied gravity—going up or staying the same but rarely coming down.”^19^

In 2006, the RUC established what is now called the Relativity Assessment Workgroup to focus on five-year reviews and misvalued codes. In 2008, in response to recommendations from MedPAC and criticism from members of Congress, CMS requested that the RUC review the relative values for fast-growing, high-cost procedures identified by CMS.^20^ CMS also identified several potential screens for misvalued codes, including the fastest growing procedure codes, codes that had not been updated since initial valuation by Harvard researchers, and direct inputs for PE RVUs. The RUC also developed its own screens such as site of service anomalies and high intraservice work per unit time.^21^

CMS also annually asks the public to nominate misvalued codes, although most public comments focus on codes that clinicians perceive to be undervalued.^22^ The highly technical nature of code valuations makes it difficult for non-clinicians, and even those in different medical specialties, to nominate potentially overvalued codes for review.

CMS' push for more attention to overvalued codes has produced some success. CMS and the RUC's efforts have focused not just on work RVUs for flagged codes, but also on the associated PE and PLI RVUs. Further, the RUC and CMS routinely assess entire families of codes, potentially extending savings from any identified overvaluations. In 2010, the misvalued code initiative generated $491 million in savings to be redistributed to the rest of the MPFS by reducing the value of some codes, particularly by bundling codes related to myocardial profusion imaging (authors' analysis of data provided by AMA staff). In 2013 and 2014, the RUC achieved redistribution of $3.1 billion, mostly from changes to direct PE in pathology, radiology, and ultrasounds (authors' analysis of data provided by AMA staff).

Congress created additional requirements and incentives for CMS to address misvalued codes in three laws enacted in 2014: the Patient Protection and Affordable Care Act, the Protecting Access to Medicare Act of 2014, and the ABLE Act of 2014.^4^ Taken together, these laws require CMS to periodically identify services that may be misvalued; develop processes to validate the accuracy of relative values; and collect data to help establish more accurate relative values. CMS was also authorized to transfer $2 million per year from the Federal Supplementary Medical Insurance Trust Fund beginning in 2014 to collect and use such data.^23^ When these funds initially became available, CMS used them to conduct feasibility studies of alternative sources of physician time data, but it is not clear if or how the funds have been spent in recent years, as the last report on use of the funds was in 2017.^24^

## Despite penalties, CMS and the RUC failed to meet modest targets

Between 2016 and 2018, the ABLE Act required CMS to reduce overvalued RVUs in the MPFS to achieve total savings of 0.5% to 1.0%, depending on the year. Missing the targets carried a significant penalty for physicians, reducing the entire MPFS by the gap between the target and CMS' actual savings from misvalued codes. This penalty was applied without consideration for budget neutrality. Despite this, CMS and the RUC missed the targets every year, lowering payment for all physicians (Table 1). Over the 2016 to 2018 period, the conversion factor was reduced a cumulative −1.04 due to this failure, reducing the pay of all physicians who bill Medicare by ∼$2 billion.

**Table 1.** ABLE act targets and reductions in RVUs, 2016-2018.

Year	ABLE act target	Actual RVU reduction	Penalty	Final CF after penalty	Estimated lost Medicare payments to physicians
*2016*	1.0%	0.23%	−0.77 CF	$35.8279	$1.5 billion
*2017*	0.5%	0.32%	−0.18 CF	$35.8887	$347 million
*2018*	0.5%	0.41%	−0.09 CF	$35.9996	$173 million

Source: CMS Physician fee schedule final rules for 2016, 2017, and 2018.^24-26^

Notes: CF is Conversion Factor. To create the “Estimated Reduction in Medicare Payments to Physicians,” we used data from the Medicare Trustee's report on Medicare fee schedule spending each year,^27-29^ excluding beneficiary cost-sharing. We divided Medicare spending by the conversion factor to arrive at an estimate of the total number of RVUs, then multiplied that estimate by the conversion factor that would have been in place absent the ABLE Act reductions.

CMS and the RUC did not miss the ABLE Act targets because there were simply too few overvalued codes, however. Two reports commissioned by CMS used direct observations and Electronic Health Record (EHR) data to estimate physician time for services and procedures, and they found systematic overvaluation of intraservice time in work RVUs.^30,31^ In 2018, in response to CMS's annual request for public nominations of misvalued services, Anthem, Inc. (now Elevance Health, Inc.) submitted a comment letter arguing that overvaluations of work were significantly larger than the 0.5% targets set by the ABLE Act.^32^ Anthem identified seven potentially overvalued services for CMS to review^32^ based on data from CMS-commissioned reports.^30^ Although CMS gave the list to the RUC for review, the RUC peremptorily reaffirmed the work values and did not recommend any significant changes in values for these codes.

One code family Anthem identified as potentially overvalued was colonoscopies, which currently represent nearly $700 million in allowed charges. In 2013, the Washington Post reported that physicians, “would have to be averaging more than 24 hours a day to perform all of the procedures that they are reporting.”^14^ The RUC revalued colonoscopies in 2016, marginally reducing the estimated physician time from about 75 to 65 minutes, reducing the estimated work about the same percentage. The estimated time and work have remained unchanged since then. Despite empirical time measurement that the procedure typically requires 30 minutes,^14,30^ the RUC and CMS continue to assume the time is more than twice that and summarily and peremptorily rejected Anthem's request for a fresh revaluation.

As of 2025, the RUC asserts that it has examined 95% of the MPFS for misvaluations and has achieved $5 billion in redistribution over a period of 17 years.^33^ While that may sound substantial, the MPFS currently pays for a little over $90 billion in allowed charges per year.^1^ Further, over this period, ∼70% of the RUC's redistribution savings come from revaluations of PE, not work (authors' analysis of data provided by AMA staff), despite extensive evidence that RUC surveys have dramatically overestimated work time for many common procedures.^30,31^

For example, there is substantial evidence of work exaggeration in treatments of warts and actinic keratoses with sprays of liquid nitrogen. These treatments take mere seconds to complete, but the RUC estimates they take 23 and 29 minutes, respectively, when recommending work values. In 2023, Medicare approved allowed charges of more than $1 billion for these two services (authors' analysis of two Centers for Medicare and Medicaid Services data sets: the Restructured BETOS Classification System and the 2022 Physician/Supplier Procedure Summary). Medicare also approved about $1 billion dollars for interpretations of electrocardiograms. The RUC estimates 6 minutes of work time for this service, while a time-and-motion study found that physicians typically spend under 15 seconds.^34^ These are egregious examples of time and work inflation, but the problem is endemic, especially for minor procedures and test/imaging interpretations.

## Why the potentially misvalued code initiative has failed to reach its potential

The RUC has long been criticized for conflicts of interest, prioritizing specialties and procedures over primary care, and failing to consider alternative data sources to value physician work.^4,30,35,36^ The RUC and CMS's failure to address misvalued codes lies, in large part, in the specialty survey process that is the basis for the RUC work RVU recommendations.

The specialty survey process creates overestimates of the time and intensity required to complete specific tasks. However, when the RUC or CMS identifies codes with potentially misvalued work, the RUC relies on specialty societies to decide whether the code is misvalued and to field a new survey if needed. If a new survey is fielded, the current value of the code is included in the survey along with a list of potential reference codes, possibly anchoring respondents to keep their estimates at or near the current level. The primary method for revaluing potentially misvalued codes uses the same survey process that created the misvaluation in the first place.

The RUC has recommended somewhat more reductions in PE RVUs than work RVUs over the past 17 years due to new technology, but the direct PE valuation process is also flawed for many codes. For major procedures, there are standard direct PE packages, but otherwise the RUC relies on expert panels to revise direct PE costs for misvalued codes. Like with the specialty society surveys, these panels involve judgement calls and inherent biases.

Although CMS often proposes lower alternative work RVUs than what the RUC recommends in its proposed rules, it rarely implements them in the final rule, citing opposition to reductions from commenters for the higher RVUs.^24-26^ Specialty societies routinely organize their members to comment on CMS proposed rules; whereas, there are no organized public comment campaign efforts focused on reducing MPFS overvaluations. This skew in comments received complicates CMS's ability to sustain its proposed work reductions in final rules.

MedPAC has long recommended that CMS collect data on physician work time from a “cohort of efficient practices” to help set RVUs,^36^ a process that could also be applied to the non-physician clinician time component of direct PE. Similarly, CMS-funded research has tested the feasibility of relying on alternative, empirical data sources, including time-and-motion studies. The results of these studies show that the RUC consistently overestimates the time needed to complete many common services.^30^  ^,31^ In general, feasibility studies have found that EHR time stamps provide reliable time data for procedures, but not for a large range of mostly ambulatory services during which clinicians commonly multitask extensively without starting and stopping the timestamps in the EHR.^12^ Straightforward time-and-motion studies, however, would be able to more accurately capture time for the services not involving a significant procedure. In addition, a series of RAND studies commissioned by CMS shows that that postservice time for procedures with bundled 10- and 90-day postprocedure global periods pays for substantially more follow-up visits than are provided.^31^

Unfortunately, to date, the RUC has been unwilling to use any empirical time data that could be gathered from these sources. Instead, the RUC holds onto the magnitude estimation approach for work, a process for which there is little accountability and substantial evidence of errors. CMS has not yet developed its own approach to estimating work RVUs with alternative data sources, despite having Congressional approval to spend up to $2 million per year to do so, although it showed renewed interest in the 2026 MPFS proposed rule.^7^

## The RUC's standards for alternative data are extremely strict, and their own surveys do not meet them

In 2008, the RUC, at the request of CMS, laid out an exacting set of requirements that datasets must meet to be considered as an alternative to specialty surveys for gathering data on physician work (Table 2).^35^ These daunting requirements include, for example, that any new data source be an “extant database,” which presumably forecloses the possibility of new data collections like time-and-motion studies. To be eligible for RUC consideration, these databases must also collect data prospectively, be geographically representative, and be representative of different practice sites, subspecialties, and patient severities. A complete list of requirements is shown in Table 2.

**Table 2.** RUC criteria for extant data for use in RBRVS recommendations.

Databases must be extant
Databases must have data integrity/reliability Must collect data prospectively. Should have the ability to identify and assess outliers—multiple procedures resulting in greater LOS; diseases with high mortality rate (LOS = 0) or extended recovery (LOS > 90), age variance (bimodal). Should have the ability to have transparency of data to compare to other databases including the RUC database. Should have the ability to audit the database. Should have the ability to collect data on all cases done by participants or for large volume procedures or E/M encounters, should have sampling criteria that are statistically valid to eliminate sampling bias. Should have current data, preferably from the last 3 to 5 years, although older sets can be used for comparison purposes.
Databases should collect time data for the procedures Time data should include, at a minimum, the skin-to-skin or intraservice time and length of stay. Additional elements may include ICU LOS and other specialty specific time factors (eg, phone calls, ventilator hours).
Databases must have the ability to unequivocally map the procedure to a CPT code and isolate the procedure from associated physician work that is otherwise billable in the same setting.
Databases must list their limitations—include what is provided and not provided with respect to the RUC database.
Databases must be representative The data should be geographically representative, eg, regionally and nationally for the specialty. The data should have various levels of patient severity. The data should have adequate practice site representation and sample size—practice sites and urban and rural representation. The data should be collected from the majority of specialties (including subspecialties) that perform the procedure or encounter. The data should be collected from either hospital/institution or individual physicians.

Source: Reproduced from MedPAC's summary of RUC Extant Database Standards.^35^

The RUC's own specialty survey process meets few of these requirements. In particular, the RUC surveys do not meet the representativeness criteria, as respondents are drawn only from specialty society members, and they often have very low response rates.^4,35^ A 2011 contractor report for MedPAC noted, “We believe the collective set of criteria developed by the RUC creates an excessively high standard that no secondary source of objective data could meet. And we do not believe that the time data collected by the specialty societies through their survey process meets the exacting requirements.”^35^ However, these criteria remain in effect 14 years later, in the face of broad criticism of the RUC process by MedPAC, GAO, many policy analysts, Congress, and now CMS. The flawed specialty survey process remains in use because the RUC, with CMS's acquiescence, have set the bar too high for any alternatives to qualify.^35^

## Recommendations

CMS and the RUC have shown that they cannot address misvaluations in the MPFS even when physician payment penalties are attached, in part because they are relying on a deeply flawed process. CMS-funded studies have found alternatives feasible, including EHR time stamps and observation studies, though increased funding would be needed to support data collection.^30,31^ If CMS regularly collected PE and time data for high spending codes, RVUs could be imputed for most codes to replace the false precision of the current RUC process.

In comment letters to CMS in 2023 and 2024, a group of former CMS and MedPAC staff responsible for fee schedule implementation suggested numerous changes to how CMS sets fees.^34,37^ These included: dramatically reducing the number of payment codes in the MPFS by grouping similar service codes to reduce valuation burdens and provide fewer opportunities for unsupported payment discrepancies; identifying and reducing overpayments for procedures with 10- and 90-day global periods; developing an automatic update process for new technology that phases in reductions in work and intensity for expected productivity gains; and implementing a “building block” approach for calculating work RVUs to replace magnitude estimation, considering time and intensity separately in a much more disciplined manner.

To accomplish this major overhaul, the commenters proposed creating an advisory panel within CMS to advise already overburdened staff on MPFS issues and priorities for change. This expert panel would not replace the RUC, which would still provide valuable expertise on the clinical details of thousands of codes.

The RUC would also continue to provide information to CMS on direct PE and physician work, as well as on operational challenges in implementing MPFS changes. If it so chose, the RUC could also work to establish a limited number of standardized intensity levels across all codes in the MPFS. If the RUC declined to undertake this activity, clinically oriented policy research units could do so. CMS could, for example, tap the expertise of federal physicians at Department of Veteran's Affairs, Department of Defense, and the National Institutes of Health, and commission contractors to collect empirical time data.

CMS would need more resources to support such a process. While the current RUC process provides extensive free labor to CMS, it comes at a high cost in misallocation of MPFS dollars. The RUC process likely costs more than it saves in unnecessary utilization and shortages in primary care and behavioral health professionals. CMS can and should do more to ensure that the MPFS becomes more accurate, fair, and effective.

## Supplementary Material

qxaf189_Supplementary_Data
