# Supplementary material for: Why the Medicare physician fee schedule misvalues fee levels and how to fix it
Source: Health Aff Sch. 2025 Oct 1;3(10):qxaf189. doi: 10.1093/haschl/qxaf189 (PMC12508800; doi:10.1093/haschl/qxaf189)
Supplement: qxaf189_Supplementary_Data [file qxaf189_supplementary_data.zip › Appendix.docx]

**Appendix: A Primer on the Resource-Based Relative Value Scale and Relative Value Units**

Prior to 1992, Medicare paid physicians based on their historical charges, leading to rapid fee inflation. In 1992, Medicare adopted the Resource-Based Relative Value Scale (RBRVS), which pays physicians based on the estimated relative resources required to provide thousands of different medical services. CMS uses the RBRVS to assign a weight for each service based on the resources needed to provide the service.

There are three relative value resource categories that underlie the Medicare physician fee schedule: physician work, practice expense, and practice liability expense. The fee schedule payment for a service equals the sum of the three categories, expressed in relative value units (RVUs), adjusted for payment locality input cost differences and multiplied by a national conversion factor that translates RVUs into dollars. Congress requires that any updates to RVUs be budget-neutral, meaning increases in RVUs for some codes are offset by reductions in the overall conversion factor, thereby reducing the fees for the other services.

The RVUs for physician work represent the physician’s time and the intensity of the service. The original work RVUs in the fee schedule were developed by researchers at Harvard relying on physician surveys, but they have been updated over time by the AMA/Specialty Society Relative Value Scale Update Committee (RUC) and CMS. RVUs for practice expenses represent direct costs of providing services such as nonphysician labor, medical equipment, and medical supplies, as well as indirect expenses such as office rent. Finally, professional liability insurance RVUs represent the per-service costs of malpractice liability insurance.

See Berenson, Robert A., and Kevin J. Hayes. 2024. “The Road to Value Can’t Be Paved with a Broken Medicare Physician Fee Schedule.” *Health Affairs* 43 (7): 950–58. <https://doi.org/10.1377/hlthaff.2024.00299>.
